# Progress in the Enzymology of the Mitochondrial Diseases of Lipoic Acid Requiring Enzymes

**DOI:** 10.3389/fgene.2020.00510

**Published:** 2020-05-21

**Authors:** John E. Cronan

**Affiliations:** B103 Chemical and Life Sciences Laboratory, Departments of Microbiology and Biochemistry, University of Illinois at Urbana-Champaign, Urbana, IL, United States

**Keywords:** lipoic acid, lipoate assembly, pyruvate dehydrogenase, α-ketoglutarate dehydrogenase, glycine cleavage system, LIPT1, LIPT2, LIAS

## Abstract

Three human mitochondrial diseases that directly affect lipoic acid metabolism result from heterozygous missense and nonsense mutations in the *LIAS*, *LIPT1*, and *LIPT2* genes. However, the functions of the proteins encoded by these genes in lipoic acid metabolism remained uncertain due to a lack of biochemical analysis at the enzyme level. An exception was the LIPT1 protein for which a perplexing property had been reported, a ligase lacking the ability to activate its substrate. This led to several models, some contradictory, to accommodate the role of LIPT1 protein activity in explaining the phenotypes of the afflicted neonatal patients. Recent evidence indicates that this LIPT1 protein activity is a misleading evolutionary artifact and that the physiological role of LIPT1 is in transfer of lipoic acid moieties from one protein to another. This and other new biochemical data now define a straightforward pathway that fully explains each of the human disorders specific to the assembly of lipoic acid on its cognate enzyme proteins.

## Introduction

Lipoic acid is an enzyme cofactor that plays a key role in mitochondrial metabolism. All of the mammalian proteins involved in lipoic acid assembly and utilization are located in the mitochondria. The cofactor is covalently attached to subunits of enzymes involved in central pathways of mitochondrial energy and carbon metabolism and thus defects in the synthesis and attachment of lipoic acid have dire effects on human health (Mayr et al., 2014; Tort et al., 2016).

## Disease Manifestations

Neonatal patients suffering from mutations in the genes encoding the enzymes of lipoate assembly show defective respiration, accumulation of toxic levels of specific amino acids, and generally die early in life (Mayr et al., 2014; Tort et al., 2016). Note that this review will not discuss the clinical aspects of these diseases but will focus on the biochemistry of lipoic acid assembly. Reviews that discuss the diagnosis and treatment of these disorders are: (Mayr et al., 2014; Tort et al., 2016). Mutations that result in iron–sulfur cluster biosynthesis deficiencies that affect the activity of lipoic acid synthase (LIAS) (Table 1), the enzyme that inserts the lipoate sulfur atoms, will not be discussed because their effects are not specific to lipoic acid synthesis; other key enzymes (e.g., complexes II and III of the respiratory chain) are also affected.

**TABLE 1:** The nonenclature differences in *E. coli, B. subtilis* and humans.

**Protein**	***E. coli***	***B. subtilis***	***H. sapiens***
Lipoyl Synthase	LipA	LipA	LIAS
Octanoyl-transferase	LipB	LipM	LIPT2
Glycine H Protein	GcvH	GcvH	GCSH
Amidotransferase	none	LipL	LIPT1
Lipoate ligase	LiplA	LipJ	none

The key enzymes that require lipoic acid for activity are pyruvate dehydrogenase, the enzyme required for entry of carbon into the citric acid cycle and α-ketoglutarate dehydrogenase, an enzyme at the midpoint of the citric acid cycle plus the glycine cleavage system. Two other dehydrogenases, the branched chain keto acid dehydrogenase required for degradation of branched-chain amino acids and the α-oxo(keto)adipate dehydrogenase of lysine degradation also require lipoic acid for activity. However, although accumulation of branched-chain amino acids and lysine in body fluids of patients have been reported (Mayr et al., 2011, 2014), enzymological studies have generally focused on the pyruvate and α-ketoglutarate dehydrogenase due to their key roles in energy production. The loss of these two dehydrogenases short-circuits the citric acid cycle, resulting in severe respiratory deficiency and extreme muscle weakness (Mayr et al., 2014; Tort et al., 2016). Lack of glycine cleavage activity additionally results in elevated brain glycine levels which can result in a host of neurological disorders, including neurodegeneration, encephalopathy, and neonatal-onset epilepsy (Mayr et al., 2014; Tort et al., 2016; Stowe et al., 2018). In general, the first indicator of defective lipoic acid metabolism is the presence of very high levels of lactate (resulting from reduction of the pyruvate that accumulates due to loss pyruvate dehydrogenase activity) in urine and other bodily fluids. Subsequent measurement of body fluid glycine levels consigns the patients into two classes (Tort et al., 2016). Normal glycine levels demonstrate that the glycine cleavage system is functional whereas abnormally high glycine levels indicate that glycine cleavage is defective. Tissue fibroblasts (and/or liver biopsy tissue) derived from the patients are then assayed for the cognate enzyme activities plus the levels of lipoylation of the cognate enzyme proteins. Patients that display absent or low lipoylation of all three enzymes have mutations in either of two genes *LIAS* or *LIPT2* whereas patients that retain glycine cleavage activity are mutant in a third gene, *LIPT1* (Mayr et al., 2014; Tort et al., 2016; Table 1). The phenotypes of the two classes of patients could be explained by a simple model in which LIAS and LIPT2 are required for lipoic acid synthesis whereas LIPT1 is not required (demonstrated by the normally lipoylated glycine cleavage system H protein (GCSH) of *LIPT1* patients). Since it was clear that LIAS was the sulfur insertion enzyme (Mayr et al., 2011), LIPT2 could be assigned a role in providing the substrate of LIAS whereas LIPT1 would function to relay lipoyl groups from GCSH to the pyruvate and α-ketoglutarate dehydrogenases. (Note that the protein products of the genes have the same name as the encoding gene except that protein names lack italics.) However, there was no precedent for such a pathway in nature and the LIPT1 protein had an enzymatic activity that muddled this simple model. It now seems clear that this misleading enzyme activity is an evolutionary artifact (see below).

### The Pathway of Lipoic Acid Assembly and Attachment to the Cognate Proteins

**FIGURE 1:**
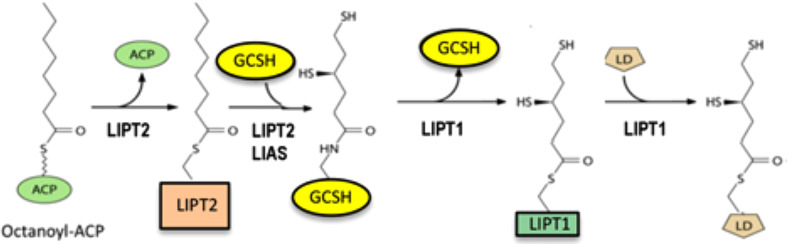
The human pathway of assembly of lipoyl moieties on its cognate proteins. LIPT2 utilizes octanoyl-ACP synthesized by the mitochondrial fatty acid synthesis system to modify GCSH. The reaction proceeds via a LIPT2 acyl enzyme intermediate as shown. The octanoyl-GCSH is then converted to lipoyl-GCSH by LIAS catalyzed sulfur insertion. LIPT1 then transfers the lipoyl moiety from GCSH to form an acyl enzyme intermediate. This in turn is attacked by a specific lysine reside of the lipoyl domain (LD) of the N-termini of the E2 subunits of the dehydrogenases or GCSH to give the active modified enzymes.

The background of the lipoic acid assembly and attachment pathway is required to place the products of these genes in context. In bacteria *de novo* synthesized lipoic acid attached to its cognate enzyme proteins (lipoyl modification) was shown to arise by a markedly unusual pathway: lipoyl groups are assembled on the cognate enzyme proteins (Figure 1). Although this was first uncovered by genetic and biochemical analysis in bacteria (see below), the bacterial and human proteins are members of the same protein family (see below). The “backbone” of lipoic acid is octanoic acid, an eight-carbon fatty acid synthesized by a distinct mitochondrial fatty acid synthesis system. The relevant form of octanoic acid is that attached to a small carrier protein of fatty acid synthesis called acyl carrier protein (ACP). The chemical linkage that couples octanoate to ACP greatly facilitates transfer of the octanoyl group to the glycine cleavage H protein (GCSH) where the covalently bound octanoyl groups are converted to lipoyl groups by insertion of the two sulfur atoms required for function of the cognate enzymes. Following sulfur insertion lipoyl groups are relayed to the E2 subunits of the two dehydrogenases. The human pathway of Figure 1 has been validated by direct enzyme assays of the proteins and their activity in a reconstituted human pathway (Cao et al., 2018). Hence GCSH functions both in glycine cleavage and in assembly of lipoyl moieties. Indeed, bacterial strains that lack GCSH are defective in both glycine cleavage and lipoic acid assembly (Christensen et al., 2011; Cao et al., 2018).

### The Pathway Explains the Metabolic Defects Seen in Neonatal Patients

Given the pathway (Figure 1) we can now interpret the biochemical phenotypes of the patients. LIPT2 and LIAS proteins are required for lipoic acid assembly because LIPT2 is the enzyme that transfers octanoyl groups from ACP to GCSH to provide the substrate for sulfur insertion by LIAS. Hence patients defective in either of these genes have undetectable or severely decreased levels of all lipoylated proteins and undergo the severe respiratory deficiency and extreme muscle weakness due to citric acid cycle disfunction plus the neurological impairments caused by glycine accumulation in the brain (Tort et al., 2016). In contrast LIPT1 patients generally suffer only the respiratory and muscle weakness problems because GCSH is lipoylated and glycine is cleaved (Tort et al., 2016). The two dehydrogenase proteins lack lipoylation because LIPT1 is the enzyme that transfers lipoyl groups from lipoyl-GCSH to the dehydrogenase proteins. Hence, glycine cleavage is normal and most of these patients escape the neurological problems resulting from glycine accumulation. However, there are reports of neurological involvements in LIPT1 patients (Soreze et al., 2013; Tache et al., 2016; Stowe et al., 2018) which remain unexplained.

### The Misleading Defective Ligase Activity of the LIPT1 Protein

Beginning in 1969 Japanese investigators began study of lipoic acid attachment to the glycine cleavage H protein of various vertebrates (Fujiwara et al., 1994, 1996, 1997, 2001). They identified a puzzling enzyme encoded the by the human *LIPT1* gene that could transfer the activated intermediate lipoyl-AMP to the H protein. However, the origin of lipoyl-AMP remained a mystery. Since classical ligases in general use ATP to activate the substrate to be ligated as well as perform the transfer of the substrate to the acceptor molecule, the protein seemed a peculiar “half ligase” unable to activate its substrate. This was in contrast to an early report that lipoate was readily attached to pyruvate dehydrogenase in an ATP-requiring reaction that proceeded through a lipoyl-AMP intermediate (Reed et al., 1958) in bacterial extracts. However, since the preparations were only partially purified, the activity could have been due to more than one protein. In 1994 the first homogenous bacterial lipoate ligase was reported (the LplA of *E. coli*) and unlike the mammalian and avian proteins, it performed the full ligase reaction: incubations containing lipoate and ATP resulted in formation of lipoyl-AMP and the lipoyl moiety was then transferred to the cognate acceptor proteins with release of AMP (Morris et al., 1994). Curiously, this *E. coli* protein aligned readily with the vertebrate LIPT1 proteins except for the C-terminal 25% of the proteins at which point the sequences diverged greatly (Fujiwara et al., 2001). Subsequent crystal structures of the *E. coli* LplA and bovine LIPT1 proteins showed that although the N-terminal 75% of the protein structures could be superimposed, the two C-terminal domains were positioned very differently (Fujiwara et al., 2007, 2010). Subsequent studies of other lipoate ligases indicated that the C-terminal domain is required for lipoyl-AMP synthesis consistent with the lack of this activity in the vertebrate proteins (Christensen and Cronan, 2009; Cao and Cronan, 2015). Despite the inability of the vertebrate proteins to perform the overall ligase reaction, several investigators assigned it a role in scavenging dietary lipoate which argued that feeding lipoate to patients should bypass genetic defects in lipoate assembly.

### Insights From Studies in Mammals, Yeast and Bacteria

The first indication that lipoate supplementation was unable to reverse the genetic defects in lipoic acid metabolism came from studies of *LIAS* knockout mice (Yi and Maeda, 2005). Despite supplementation of the diets of the heterozygous mothers with lipoic acid during pregnancy, all homozygous *LIAS* null embryos died *in utero* and were reabsorbed This was consistent with the findings that lipoic acid supplementation of the diets of human *LIAS, LIPT1*, and *LIPT2* patients or of their fibroblast cultures failed to alleviate the physiological and biochemical effects of the mutant genes (Baker et al., 2014; Tort et al., 2014, 2016; Tsurusaki et al., 2015). Lipoate is known to rapidly enter the bloodstream (Teichert et al., 2003) and thus it should have been available if a fully competent ligase existed. Additional mouse and tissue culture studies also clearly demonstrated the inability of supplemented lipoic acid to become attached to the key lipoic acid modified proteins of central metabolism (Witkowski et al., 2007; Feng et al., 2009; Smith et al., 2012).

Two genetic selections gave *E. coli* mutants devoid of ligase activity due to disruption of the encoding gene, *lplA*. These selections were done based on the premise that the ligase played a role in lipoic acid synthesis but this soon found not to be the case. The mutant bacteria had normal levels of lipoylated enzymes and grew normally (Morris et al., 1994). Further work showed that the physiological function of *E. coli* lipoate ligase (LplA) was to scavenge lipoic acid from the environment, an advantage for an enteric bacterium. These data led to a new hypothesis in which octanoyl-ACP was the substrate for sulfur insertion. Prior studies had isolated mutants that required lipoic acid for growth and these fell into two classes, those lacking the sulfur insertion enzyme (LipA, a homolog of LIAS) and an enzyme (LipB, a homolog of LIPT2) (Table 1) that transferred either lipoic acid or octanoic acid from their ACP thioesters to pyruvate dehydrogenase (Cronan, 2016). Although study of these enzymes led to the first synthesis of lipoic acid *in vitro*, (Miller et al., 2000) the putative lipoyl-ACP reaction intermediate could not be detected. The reason for this result became clear when LipB mutants were found to grow with octanoic acid supplementation in place of the usual lipoic acid supplementation (Zhao et al., 2003). Moreover, octanoate-dependent growth required LplA ligase activity. Hence, the combination of octanoate supplementation and ligase activity bypassed the lack of LipB transferase activity (Zhao et al., 2003). The most straightforward interpretation of these results was that lipoyl moieties were assembled on the cognate enzyme proteins. That is, octanoyl groups became covalently attached to the dehydrogenase and were then converted to lipoyl moieties by the LipA sulfur insertion enzyme. Hence there was no lipoyl-ACP intermediate to be detected! This “on-site” assembly was a striking departure from the usual pathway of other attached cofactors (e.g., biotin) which are fully assembled and then transferred onto the cognate protein. The pathway of lipoyl-protein assembly was quickly validated by *in vivo* and *in vitro* studies in several laboratories (Booker, 2004; Cicchillo et al., 2004; Bryant et al., 2006; Douglas et al., 2006). Hence *E. coli* lipoyl assembly requires only two enzymes, the LipB octanoyl transferase and the sulfur insertion enzyme, LipA.

Lipoic acid assembly has also been studied in the yeast, *Saccharomyces cerevisiae* mainly by genetic studies and immunological assays of protein lipoylation (Sulo and Martin, 1993; Schonauer et al., 2008). The picture that emerged is consistent with the human pathway (see below). However, the yeast proteins involved in lipoate assembly form intractable inclusion bodies when expressed in *E. coli* or in a yeast expression system (Hermes and Cronan, 2013). Hence minimal *in vitro* enzymology has been done and that was done with proteins refolded from inclusion bodies, a serious caveat (Hermes and Cronan, 2013). Given the lack of direct demonstrations of the enzymatic activities of the proteins, the yeast pathway lacks the biochemical grounding of the human pathway. An unexplained facet is that most of the yeast lipoic acid synthesis mutants were originally isolated as strains defective in processing of mitochondrial RNAs (Sulo and Martin, 1993; Schonauer et al., 2008).

### The Human Pathway Is Remarkably Similar to That Used by Gram-Positive Bacteria and Probably by Yeast

The gram-positive bacterium, *Bacillus subtilis*, was studied because it lacked a gene that encoded a recognizable LipB-like octanoyl transferase although the bacterium had a *lipA* gene, disruption of which resulted in a growth requirement for lipoic acid (Martin et al., 2009). The ability to use exogenous lipoic acid argued that the bacterium must have a functional lipoate ligase. However, analysis of the *B. subtilis* genome showed not one but three genes encoding putative lipoate ligases, albeit they had only low sequence identity with *E. coli* LplA. One of these genes encoded a ligase (LplJ) that functionally replaced LplA in *E. coli* (Martin et al., 2011) whereas a second putative ligase gene encoded an octanoyl transferase (LipM) that had no ligase activity (Christensen and Cronan, 2010). The protein encoded by third ligase candidate gene, LipL, also lacked ligase activity and seemed an enigma, although its inactivation resulted in a growth requirement for lipoic acid. A series of biochemical experiments with purified proteins presented the perplexing result that the *B. subtilis* LipM octanoyl transferase was unable to transfer octanoate from octanoyl-ACP to its native pyruvate dehydrogenase E2 subunit, although it was active with a foreign (*E. coli*) pyruvate dehydrogenase subunit (Christensen et al., 2011). These results argued that a factor or factors was missing. Two were found: GcvH, the GCSH and the enigmatic third ligase candidate, LipL (Christensen et al., 2011). Biochemical analyses showed that the LipM octanoyl transferase attached an octanoyl moiety to GcvH which LipA converted to a lipoyl group. LipL then transferred the lipoyl group from GcvH to the dehydrogenase proteins (Christensen et al., 2011). This was the first biochemical evidence for “lipoyl relay,” the process used in the human and yeast pathways. Indeed, the human pathway reaction scheme given in Figure 1 can describe that of *B. subtilis* given substitution of LipM for LIPT2, GcvH for GCSH, and LipL for LIPT1.

## Discussion

### Evolutionary Considerations

Why is lipoic acid assembled on its cognate enzymes rather than on octanoyl ACP? A good rationale is economy: the levels of cognate proteins to be modified dictate that the cell can make only the amount of lipoic acid required; no excess is made.

Is there an explanation for why lipoyl moieties are relayed from the glycine cleavage H protein to the dehydrogenase rather than directly assembled on the dehydrogenases? A persuasive argument put forth by Braakman and Smith (2014) is that lipoyl relay seems be a “make-do” device that arose when primitive bacteria having the glycine cleavage H protein as the sole lipoylated protein acquired lipoate-requiring dehydrogenases.

All of the enzymes that transfer octanoate or lipoate are related by their structures but with little sequence conservation and comprise a protein family (Pfam PF03099). Crystal structures show that the lipoate ligases (LplA, LplJ), the octanoyl transferases (LIPT2, LipB, LipM), and the lipoyl relay enzymes (LIPT1 and LipL) are all constructed on the same structural scaffold albeit with only minimal amino acid sequence conservation (generally 15–25%) (Cronan, 2016). Sometimes the scaffold has given rise to the same enzyme activity by seemingly different routes. An example is the two bacterial octanoyl transferases (LipB and LipM) which have their active site cysteine residues lie on different loops of the scaffold (Christensen and Cronan, 2010). The Pfam PF03099 scaffold is also found in the biotin protein ligases that attach biotin to its cognate proteins. The fact that proteins having similar sequences (e.g., LplA and LIPT1) can have different enzyme activities makes assignment of functions based solely on sequence comparisons perilous.

Why has LIPT1 retained a partial lipoate ligase reaction when it clearly functions as an amidotransferase that transfers lipoate from GCSH to the dehydrogenases E2 subunits? The answer seems to lie in a newly appreciated aspect of enzyme evolution called “moonlighting”; a protein can acquire a new enzymatic activity while fully or partially retaining its original activity (Copley, 2012, 2014; Jeffery, 2018, 2019). That is, mutations can enhance the moonlighting function without concomitant elimination of the ancestral function. The original ancestor of LIPT1 seems likely to have been a fully functional lipoate ligase like LplA encoded by the genome of the bacterial endosymbiont that became the mitochondrion. In this scenario the price for retooling the ligase into an amidotransferase was that the carboxyl terminus lost the ability to activate lipoate to lipoyl-AMP and the protein became a “half-ligase” and (temporarily?) retained the partial ligase activity. In other words, the lipoyl-transfer activity is an evolutionary remnant, an aspect often seen in moonlighting proteins (Copley, 2014; Jeffery, 2018). Note that moonlighting as been observed for medically important proteins (Sriram et al., 2005; Houten, 2017) and the possibility that moonlighting could complicate understanding of single gene Mendelian inherited disorders has been raised (Sriram et al., 2005; Franco-Serrano et al., 2018). Indeed, the partial LIPT1 ligase activity markedly hindered our understanding the human pathway of lipoyl-protein assembly.

### The Future?

Although the biochemistry and physiology of the human disorders of lipoic acid metabolism is now understood this knowledge offers few approaches to ameliorate the suffering of these patients (as is the case with other mitochondrial diseases). At present this reviewer can offer only the remote possibility of introducing a bacterial ligase into the mitochondria perhaps by inserting a modified ligase gene into the nuclear genome. The encoded ligase would carry an N-terminal sequence targeting it to the mitochondria. The *E. coli* lipoate ligase is known to modify human lipoyl enzymes (Quinn, 1997).

## Author Contributions

JC wrote the entire manuscript and takes full responsibility for the contents.

## Conflict of Interest

The authors declare that the research was conducted in the absence of any commercial or financial relationships that could be construed as a potential conflict of interest.
